# Supplementary material for: Enrichment of circulating trophoblasts from maternal blood using filtration-based Metacell® technology
Source: PLoS One. 2022 Jul 14;17(7):e0271226. doi: 10.1371/journal.pone.0271226 (PMC9282611; doi:10.1371/journal.pone.0271226)
Supplement: S1 Fig — All calibrator samples consist of male DNA in a background of 70 ng female DNA. (DOCX) [file pone.0271226.s001.docx]

**S1 Fig. Standard curve, R², and equation for Y-qPCR analysis.** All calibrator samples consist of male DNA in a background of 70 ng female DNA.
