# Supplementary figures and images for: Enrichment of circulating trophoblasts from maternal blood using filtration-based Metacell® technology
Source: PLoS One. 2022 Jul 14;17(7):e0271226. doi: 10.1371/journal.pone.0271226 (PMC9282611; doi:10.1371/journal.pone.0271226)

**S2 Fig. Y-STR profile of a calibrator sample containing 10 pg male DNA in a background of 70 ng female DNA.**

**
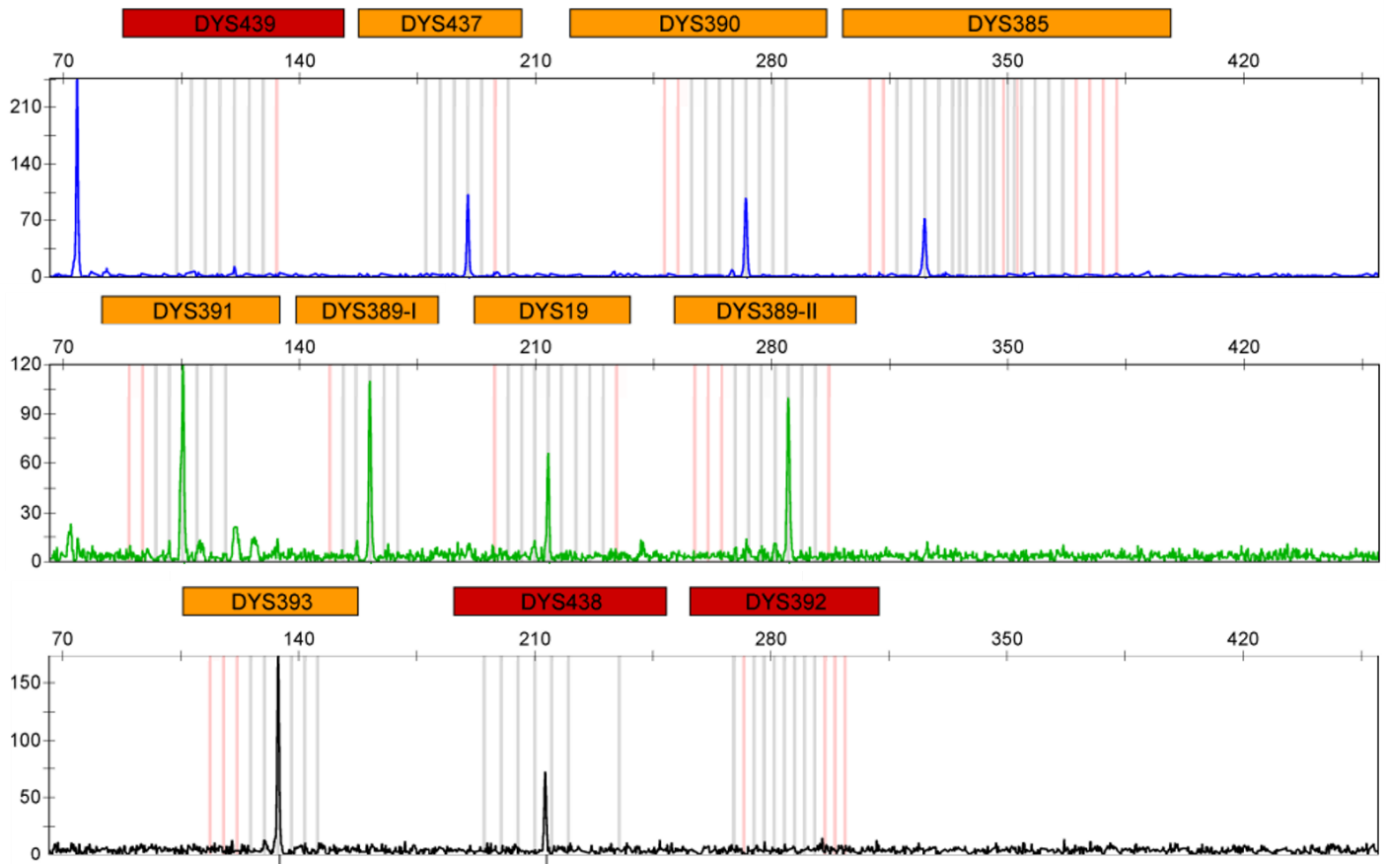
**

Supplement: S2 Fig — (DOCX) [file pone.0271226.s002.docx]
