# Supplementary material for: Enrichment of circulating trophoblasts from maternal blood using filtration-based Metacell® technology
Source: PLoS One. 2022 Jul 14;17(7):e0271226. doi: 10.1371/journal.pone.0271226 (PMC9282611; doi:10.1371/journal.pone.0271226)

**S3 Fig. Y-STR profile of sample 6, 12, 19, and 22.** An allele is called if the fluorescent signal is higher than 50 RFU. Called alleles are marked with a blue arrow.


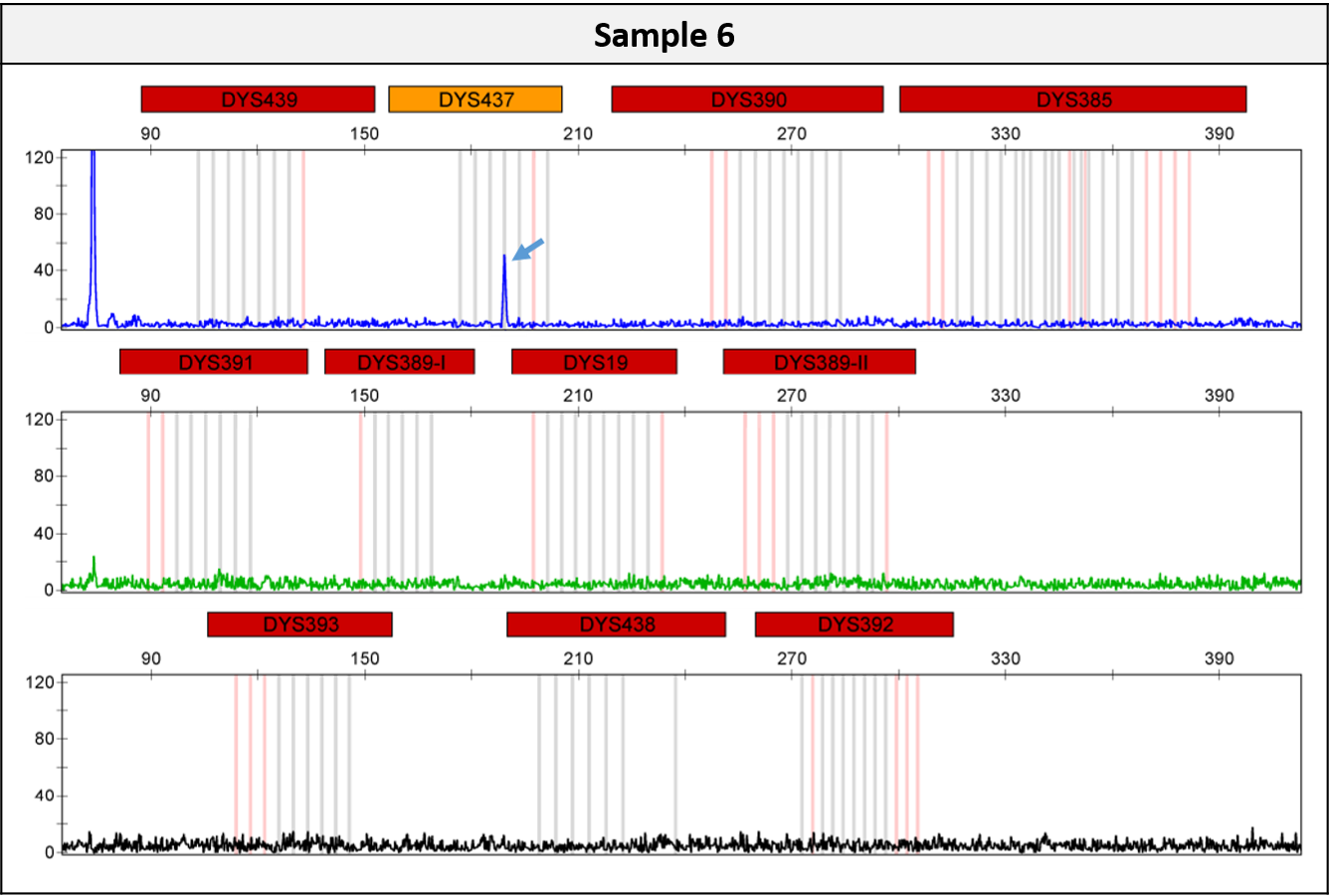


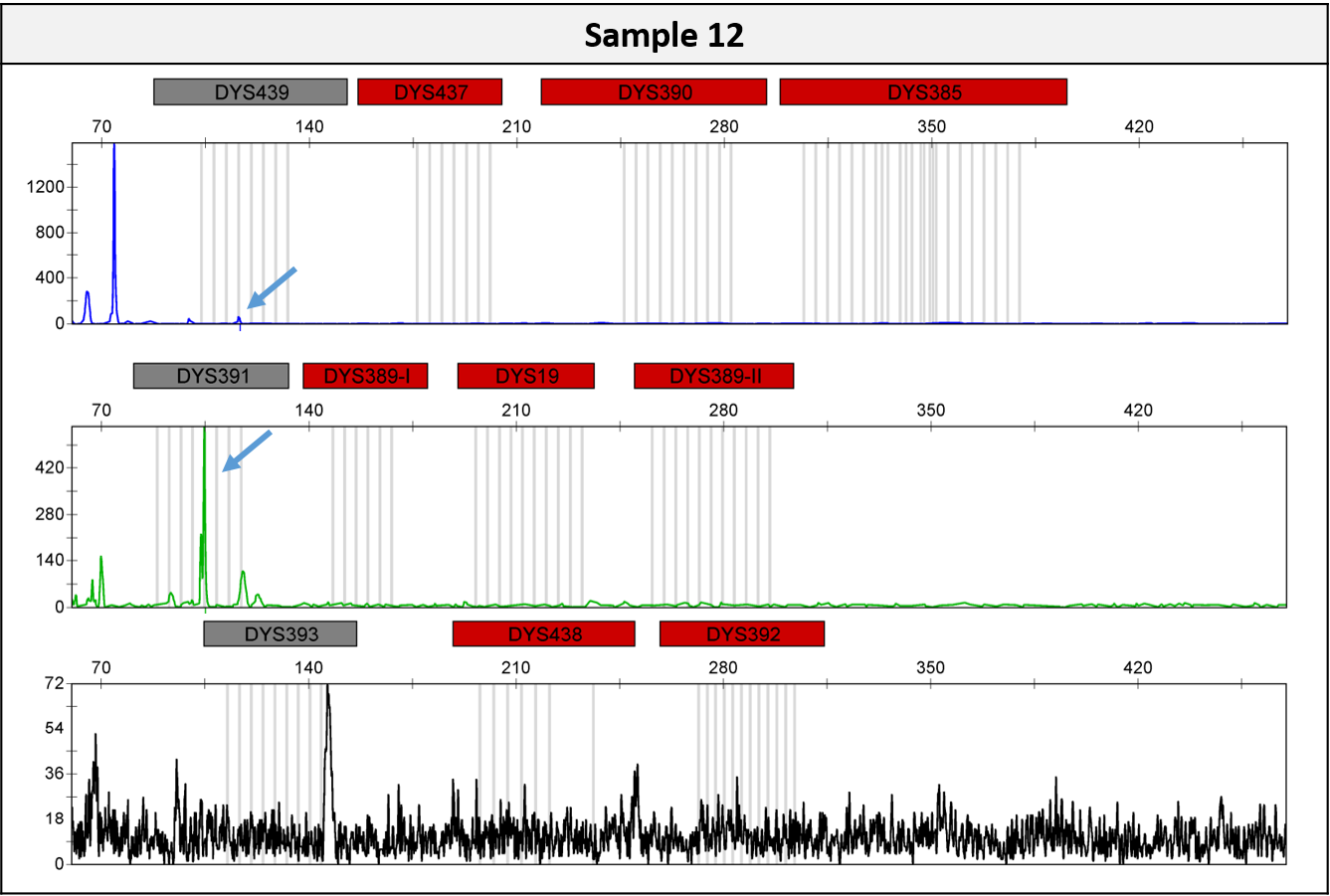


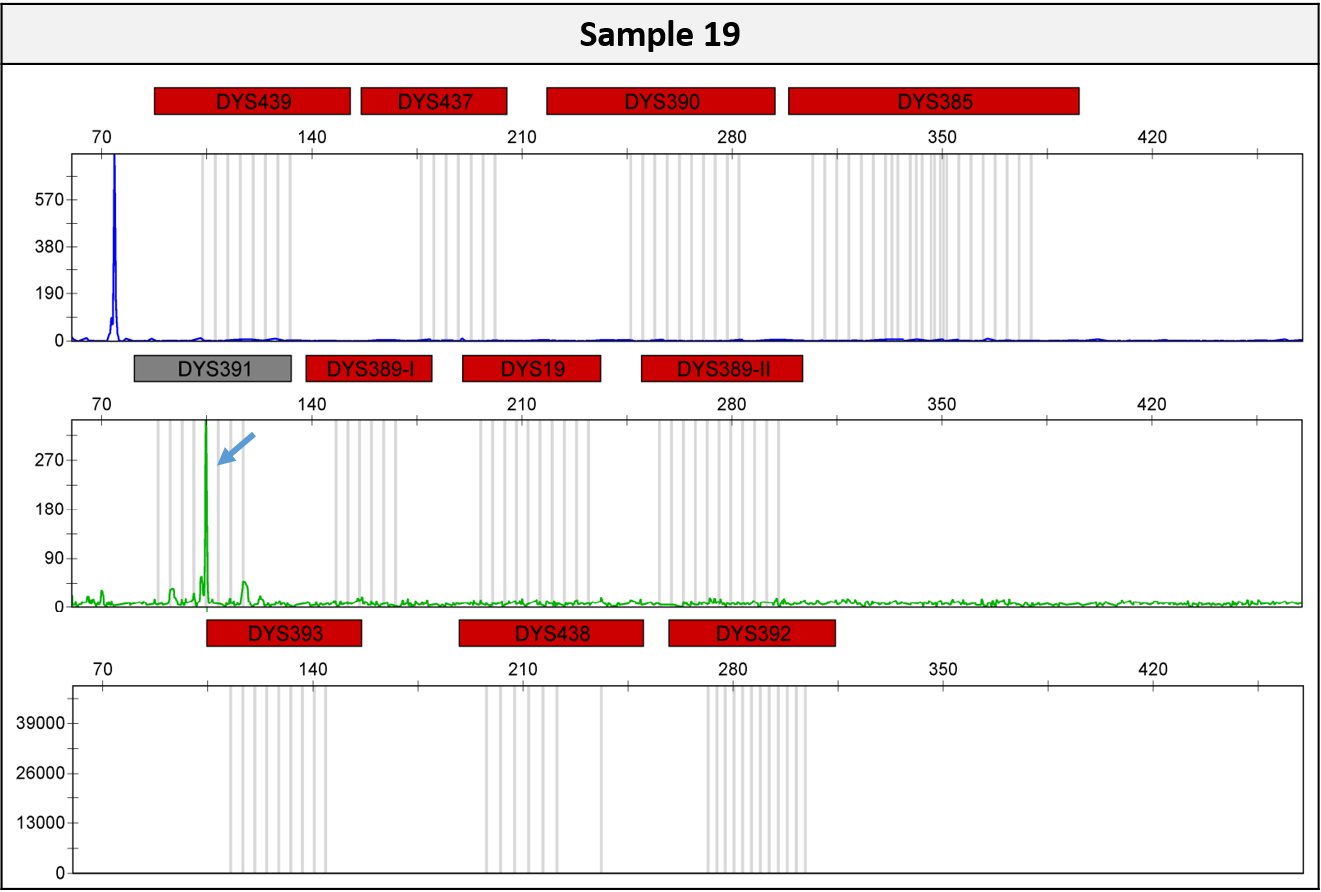


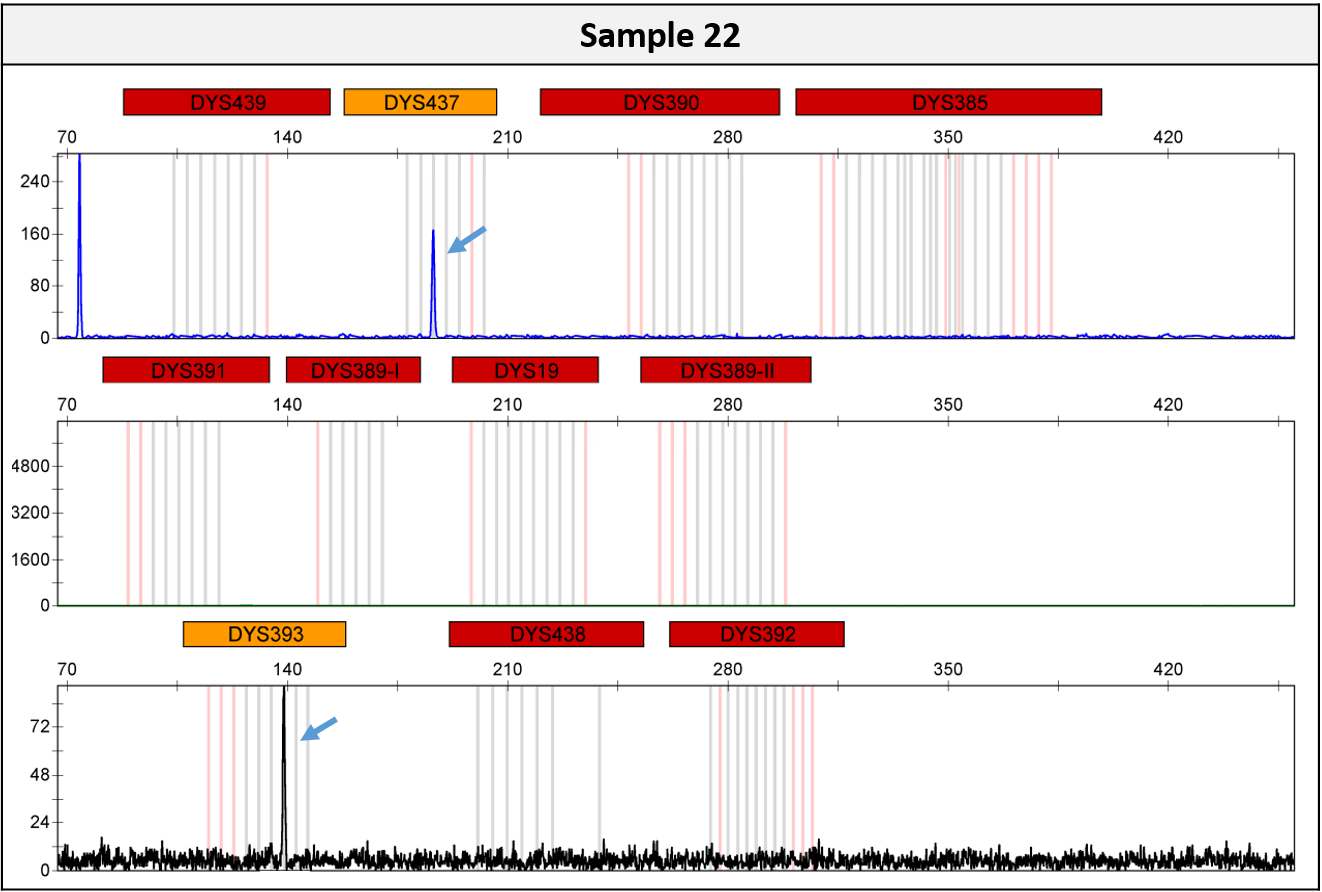

Supplement: S3 Fig — An allele is called if the fluorescent signal is higher than 50 RFU. Called alleles are marked with a blue arrow. (DOCX) [file pone.0271226.s003.docx]
