# Supplementary material for: Enrichment of circulating trophoblasts from maternal blood using filtration-based Metacell® technology
Source: PLoS One. 2022 Jul 14;17(7):e0271226. doi: 10.1371/journal.pone.0271226 (PMC9282611; doi:10.1371/journal.pone.0271226)
Supplement: S1 Table — (DOCX) [file pone.0271226.s004.docx]

**S1 Table. Plasma C_q_ values with corresponding male DNA concentration, and fetal sex confirmation by Ghent University Hospital.**

| **Sample** | **Plasma C_q_ triplicates** | | | **Average plasma C_q_** | **Male DNA concentration in plasma (pg/µL)*** | **Fetal sex confirmed by Hospital** |
| --- | --- | --- | --- | --- | --- | --- |
| 1 | 29.47 | 29.64 | 29.41 | 29.51 | 2.45 | Male |
| 2 | 29.68 | 30.06 | 29.91 | 29.89 | 2.01 | Male |
| 3 | 30.21 | 30.38 | 30.20 | 30.26 | 1.65 | Male |
| 4 | / | / | / | / | / | Male |
| 5 | / | / | / | / | / | Male |
| 6 | 29.41 | 29.60 | 29.62 | 29.54 | 2.40 | Male |
| 7 | / | / | / | / | / | Male |
| 8 | / | / | / | / | / | Male |
| 9 | / | / | / | / | / | Male |
| 4 | / | / | / | / | / | Male |
| 11 | / | / | / | / | / | Male |
| 12 | / | / | / | / | / | Male |
| 13 | 27.33 | 27.31 | 27.31 | 27.32 | 7.70 | Male |
| 14 | 30.79 | 30.34 | 30.57 | 30.57 | 1.41 | Male |
| 15 | 30.67 | 30.46 | 30.74 | 30.62 | 1.37 | Male |
| 16 | / | / | / | / | / | Male |
| 17 | 30.62 | 30.73 | 30.81 | 30.72 | 1.30 | Male |
| 18 | 29.97 | 30.04 | 29.96 | 29.99 | 1.90 | Male + Female |
| 19 | / | / | / | / | / | Male |
| 20 | 29.30 | 29.43 | 29.37 | 29.37 | 2.63 | Male |
| 21 | 28.74 | 28.92 | 28.66 | 28.77 | 3.60 | Male |
| 22 | 28.73 | 28.57 | 28.63 | 28.64 | 3.85 | Male |
| 23 | 29.46 | 29.55 | 29.66 | 29.56 | 2.38 | Male |
| 24 | 29.76 | 29.54 | 29.62 | 29.64 | 2.28 | Male |
| 25 | 28.84 | 28.87 | 28.91 | 28.87 | 3.41 | Male |
| 26 | 27.67 | 27.60 | 27.52 | 27.60 | 6.65 | Male |
| 27 | > 35 | > 35 | > 35 | > 35 | / | Female |
| 28 | > 35 | > 35 | > 35 | > 35 | / | Female |
| 29 | > 35 | > 35 | > 35 | > 35 | / | Female |
| 30 | > 35 | > 35 | > 35 | > 35 | / | Female |
| 31 | / | / | / | / | / | Female |
| 32 | / | / | / | / | / | Female |
| 33 | / | / | / | / | / | Female |
| 34 | / | / | / | / | / | Female |
| 35 | > 35 | > 35 | > 35 | > 35 | / | Female |
| 36 | / | / | / | / | / | Female |
| 37 | > 35 | > 35 | > 35 | > 35 | / | Female |
| 38 | > 35 | > 35 | > 35 | > 35 | / | Female |
| 39 | / | / | / | / | / | Female |
| 40 | / | / | / | / | / | Female |
| 41 | / | / | / | / | / | Female |
| 42 | / | / | / | / | / | Female |
| 43 | > 35 | > 35 | > 35 | > 35 | / | Female |
| 44 | / | / | / | / | / | Female |
| 45 (Mixed 1) | > 35 | > 35 | > 35 | > 35 | / | Female |
| 46 (Mixed 2) | > 35 | > 35 | > 35 | > 35 | / | Female |
| 47 (Mixed 1) | 29.39 | 29.36 | 29.34 | 29.36 | 2.64 | Male |
| 48 (Mixed 2) | 29.86 | 29.90 | 29.82 | 29.86 | 2.03 | Male |
| 49 (Plasma 1) | 29.21 | 29.28 | 28.98 | 29.16 | 2.94 | Male |
| 50 (Plasma 2) | 28.67 | 28.74 | 28.70 | 28.71 | 3.73 | Male |
| 51 (Plasma 3) | 29.66 | 29.65 | 29.51 | 29.61 | 2.32 | Male |
| 52 (Plasma 4) | 27.75 | 29.55 | 29.83 | 29.71 | 3.53 | Male |
| 53 (Plasma 5) | > 35 | > 35 | > 35 | > 35 | / | Female |
| 54 (Plasma 6) | > 35 | > 35 | > 35 | > 35 | / | Female |
| 55 (Plasma 7) | > 35 | > 35 | > 35 | > 35 | / | Female |

* Male DNA in plasma was calculated using the calibration curve as visualized in Supplementary Figure SIV. 1.

C_q_: quantification cycle.
