# Supplementary material for: Enrichment of circulating trophoblasts from maternal blood using filtration-based Metacell® technology
Source: PLoS One. 2022 Jul 14;17(7):e0271226. doi: 10.1371/journal.pone.0271226 (PMC9282611; doi:10.1371/journal.pone.0271226)
Supplement: S2 Table — (DOCX) [file pone.0271226.s005.docx]

**S2 Table. Individual C_q_ values of the calibrator samples used for Y-qPCR validation, with coefficient of variation.**

| **Sample** | **Individual C_q_ values** | | | **CV(%)** |
| --- | --- | --- | --- | --- |
| 1000 pg male + 70 ng female DNA | 28.32 | 27.80 | 27.76 | 15.43 |
| 100 pg male + 70 ng female DNA | 31.69 | 30.92 | 31.23 | 19.67 |
| 50 pg male + 70 ng female DNA | 32.70 | 31.99 | 31.87 | 21.78 |
| 20 pg male + 70 ng female DNA | 35.13 | 33.72 | 33.55 | 38.80 |
| 10 pg male + 70 ng female DNA | 35.14 | 36.08 | 34.40 | 42.23 |
| 5 pg male + 70 ng female DNA | 39.72 | 37.87 | 36.24 | 80.98 |
| 2.5 pg male + 70 ng female DNA | 40.64 | 38.74 | 37.76 | 45.82 |
| 70 ng female DNA | 39.71 | 40.29 | 41.28 | 38.61 |
| NTC (water) | > 45 | > 45 | > 45 | - |

C_q_: quantification cycle, CV: coefficient of variation, NTC: no template control, Y-qPCR: Y-chromosome-specific quantitative PCR.
