# Supplementary material for: Enrichment of circulating trophoblasts from maternal blood using filtration-based Metacell® technology
Source: PLoS One. 2022 Jul 14;17(7):e0271226. doi: 10.1371/journal.pone.0271226 (PMC9282611; doi:10.1371/journal.pone.0271226)
Supplement: S3 Table — (DOCX) [file pone.0271226.s006.docx]

**S3 Table. Individual C_q_ values of the female fetus samples and female plasma samples, categorized per protocol.**

| **Protocol** | **Sample** | **Result** |
| --- | --- | --- |
| Protocol 1 | 27 | C_q_ > 40 |
|  | 28 | C_q_ > 40 |
|  | 29 | C_q_ > 40 |
|  | 30 | C_q_ = 38.61 |
| Protocol 2 | 34 | C_q_ > 40 |
|  | 35 | C_q_ > 40 |
| Protocol 3 | 39 | C_q_ = 39.26 |
|  | 40 | C_q_ > 40 |
|  | 41 | C_q_ > 40 |
|  | Plasma 5 | C_q_ = 37.08 |
|  | Plasma 6 | C_q_ = 35.40 |
|  | Plasma 7 | C_q_ > 40 |

C_q_: quantification cycle.
