# Supplementary material for: Enrichment of circulating trophoblasts from maternal blood using filtration-based Metacell® technology
Source: PLoS One. 2022 Jul 14;17(7):e0271226. doi: 10.1371/journal.pone.0271226 (PMC9282611; doi:10.1371/journal.pone.0271226)
Supplement: Attachment — Submitted filename: Response to reviewers.docx [file pone.0271226.s007.docx]

Dear reviewers,

Thank you very much for revising our paper. We appreciate your comments and have taken them in consideration for improving our manuscript and resubmitting the paper. In the following point-by-point response, the original remarks of the reviewers are indicated in dark grey italic font.

***Reviewer: 1***

*Summary of the study:*

*In search for a biomarker-free workflow for the enrichment of circulating fetal trophoblasts (CT) from maternal blood, the authors present results from a study where they use a filtration-based technology called MetaCell to isolate fetal cells from maternal blood cells.In the study they use different amounts of blood collected from pregnant women carrying male fetuses, run the sample through the device, and confirm their presence of CTs fetal using either Y specific qPCR, or Y-STR analysis. In few cases they show the presence of male Y-chromosome. However, they don’t rule out that the presence of Y-chromosome could be due to the presence of cell-free-fetal DNA, and not necessarily CTs. The Authors conclude that MetaCell technology is not apt for isolating CTs.*

*General comments:*

*Technologies isolating fetal cells from maternal blood and using them for prenatal diagnosis are attractive with a huge potential to be disruptive. A lot of research is being done not only to find new fetal cell markers, but also to exploit their different size and morphology to isolate them from maternal blood. Though the current study presents a negative result, there are some major questions and concerns that this study either misses or avoids addressing. Here are some concerns:*

- *Even though the study claims that the presence of Y specific qPCR is evidence of the presence of circulating fetal trophoblasts, no direct evidence is provided to show that the technology isolates fetal trophoblasts, and not any other fetal cell type.*

Indeed, we agree that using Y-specific qPCR, we cannot prove that the isolated DNA originates from trophoblasts. However, since trophoblasts are the largest and most abundant type of circulating fetal cells, it is more likely to enrich trophoblasts than other smaller fetal cell types, such as nucleated RBCs and fetal leucocytes, with this size-based enrichment method. We have now added lines 94-97 in the Introduction to clarify this concern.

- *The final technology workflow for using fetal cells for prenatal diagnosis is not clear. Even if the presence of fetal cells is confirmed by qPCR, what is the roadmap for isolating individual fetal cells and using them for genetic analyses?*

The Metacell technology can be implemented in a cbNIPT workflow in line with the workflow explained in Fig 2 of the manuscript: After Metacell enrichment, larger cells remain on the separation membrane. Optionally, the membrane with cells is further cultured to deplete more unwanted cells. A fluorescent dye could then be added to the culture medium, followed by visualization of the cells on the membrane with an LCM fluorescence microscope. Based on cytomorphology, cytochemical staining, or immunostaining, a single CT is selected and isolated using LCM. The genome of the single cell is then amplified with a WGA method. Eventually, DNA analysis is performed.

- *Similarly, if not for Y specific qPCR, or STR for male pregnancies, how would the fetal cells originating from female pregnancies be confirmed?*

The goal of this study is to assess the potential of the Metacell technology to enrich fetal cells. The easiest way to prove the presence of fetal cells after enrichment, is by detecting male fetal DNA by measuring the presence of Y-chromosome DNA (using Y-specific qPCR of STR analysis). We could also use maternal blood from female pregnancies and detect fetal DNA using autosomal STR-analysis. However, the interpretation of the result would be much more challenging, as at least 50% of the autosomal STRs overlap between mother and fetus. Moreover, the limited fraction of fetal cells in the presence of maternal cells results in a huge allelic imbalance which hampers efficient amplification and detection of the minor allele fraction. So for this proof-of-concept study, we chose to only use maternal blood from male pregnancies. This is now clarified in lines 110-118 of the manuscript.

***Reviewer: 2***

*Summary of the study and general comments:*

*The authors have tested the Metacell technology to enrich cells (trophopblasts) from the conceptus, circulating in maternal blood. I 3 different set-ups, the yield was none or poor. Testing this system is relevant, and the observations are as such relevant to others working in this field. However, the discussion should include considerations on whether the test set-up has tested the system sufficiently. I.e. could larger volumes of blood have been used, could the procedure show efficient if the blood was drawn at a later time. Could the filtering be followed by another procedure to further minimize contamination by maternal cells? Perhaps the authors could think of other possibilities.*

We understand your concern. We have tested the three most promising set-ups based on the set-ups of other (successful) Metacell applications. Results reveal that the main concern is the lack of enriched fetal cells, and not the number of contaminating maternal cells. However, based on our results, an unfeasible large blood volume should be processed to get the required number of fetal cells for cbNIPT. To minimize target cell loss, we’ve also processed some maternal samples that were fixed prior to filtration. This was unsuccessful, as the filter was immediately clogged. We opt to only include maternal samples drawn at GA 10-13, as this is representative for the NIPT context, which is preferably performed at the end of the first trimester. Nevertheless, we acknowledge the limitation of this study as we know that several other set-ups could have been tested. Therefore, in this study, we have concluded that the Metacell technology is not suitable for consistent enrichment of the CTs *using the protocols as tested here*. We now discuss this in the manuscript (lines 299-303).

*Minor issues:*

- *Line 34. I recognize that the term “fetal DNA” is frequently used for the DNA originating in the conceptus, identified in the maternal blood. However, please consider using another term, as likely most of this DNA does not originate in the fetus – or at least please comment on this. Similarly, the expression cffDNA is misleading. Line 43. Similarly to the comment to line 34, it would be nice avoid calling trophoblasts “fetal cells” (or at least it would be nice to read a comment on the fact that the circulating cells identified as trophoblasts, more likely originate in the placenta than the fetus). Line 49: Same comment on “fetal DNA”.*

We agree, and explain this now in lines 44-47.

- *Line 67: “blood cells” should be replaced by a term that indicates that (some of) these cells are not normally present in the blood, e.g. “cells in the blood”.*

We changed ‘blood cells’ to ‘cells in the blood’ (lines 70-71).

- *Lines 169 and 170: Some words are missing, likely ”the cells”.*

We added ‘the enriched cells’ in line 183 of the manuscript.

- *Line 176: “supernatans” likely is misspelled.*

Thank you, we corrected it to ‘supernatant’ (line 188).

- *The figures and tables are fuzzy.*

We replaced the original figures with higher-quality figures.
